# Pyomyositis of extraocular muscle: Case series and review of the literature

**DOI:** 10.4103/0301-4738.71712

**Published:** 2010

**Authors:** Ishan G Acharya, Jitendra Jethani

**Affiliations:** Department of Ophthalmic Plastic Surgery, Orbit, Ocular Oncology and Ocular Prosthesis, Dr. Thakorbhai V. Patel Eye Institute, Vadodara, Gujarat, India; 1 Department of Paediatric Ophthalmology and Squint Surgery, Dr. Thakorbhai V. Patel Eye Institute, Vadodara, Gujarat, India

**Keywords:** Extraocular muscle, pyomyositis, *Staphylococcus aureus*

## Abstract

Pyomyositis is a primary acute bacterial infection usually caused by *Staphylococcus aureus*. Any skeletal muscle can be involved, but the thigh and trunk muscles are commonly affected. Only three cases of extraocular muscle (EOM) pyomyositis have been reported. We herein present four cases of isolated EOM pyomyositis. Three of our cases presented with acute onset of proptosis, pain, swelling and redness. One patient presented with mass in the inferior orbit for 4 months. One patient had central retinal artery occlusion on presentation. None of them had marked systemic symptoms. Computed tomography scan of all patients showed a typical hypodense rim enhancing lesion of the muscle involved. Three patients were started on intravenous antibiotics immediately on diagnosis and the pus was drained externally. Two patients underwent exploratory orbitotomy. In conclusion, it should be considered in any patient presenting with acute onset of orbital inflammation. Management consists of incision and drainage coupled with antibiotic therapy.

Pyomyositis is an acute bacterial infection occurring in the skeletal muscle. It was first described in 1885 by Scriba as an endemic disease in the tropics.[1] Development of an intramuscular abscess involving the extraocular muscles (EOM) remains an extremely rare process. It usually presents as pain, swelling, redness and movement restriction in the eye. Systemic symptoms may or may not be present. To our knowledge, only three cases of pyomyositis in the eye have been reported in the literature.[2,3] We present four cases of isolated EOM pyomyositis.

## Case Reports

### Case 1

**Figure 1:**
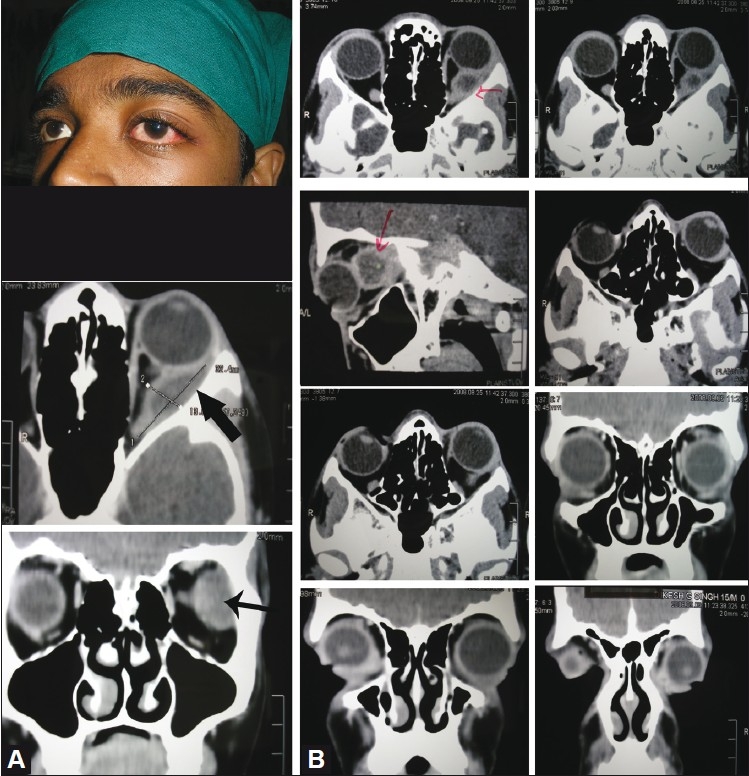
(A) (Top) left eye proptosis and semidilated pupil (middle) showing a well-defined hypodense lesion involving the left lateral rectus with rim enhancement (bottom) and compression of the mid part of the left optic nerve. (B) The uppermost two figures clearly show that the abscess is in the lateral recuts and separate from the subperiosteal plane. Other sections are for comparison

A 15-year-old boy was referred with complaints of sudden dimness of vision, proptosis, pain and swelling in the left eye (LE) for 5 days in March 2008. On examination, visual acuity in the right eye (RE) was 20/20 and in the LE was 20/200. There was proptosis of 3 mm, edema, ptosis and congestion in the superotemporal quadrant [Fig. 1A]. The pupil was semidilated (6 mm) and relative afferent papillary defect was present. Ocular motility was restricted in the superior, lateral and medial gazes. The posterior segment was normal. Computed tomography (CT) scan showed a well-defined hypodense rim-enhancing lesion involving the left lateral rectus (LR) and abutting the left optic nerve [Fig. 1A and B], with retrobulbar fat infiltration. Blood culture was negative. Differential diagnosis of LR muscle abscess or an infected orbital lesion was performed. An exploratory lateral orbitotomy was performed and pus was drained. He was put on intravenous amikacin 500 mg 12 hourly and cefotaxime 1 g 12 hourly. Vision improved to 20/30 on the very next day and the pupil became normal in size and reaction to light. Gram stain showed positive cocci and the culture report showed growth of *Staphylococcus aureus*. Ampicillin plus cloxacillin 1 g 12 hourly and amikacin were started according to the sensitivity report. He was discharged with gatifloxacin twice daily orally. After 1 month, he had residual restriction in motility in medial and superior gazes and 1 mm of proptosis. After 1 year, there was no proptosis and motility restriction.

### Case 2

**Figure 2:**
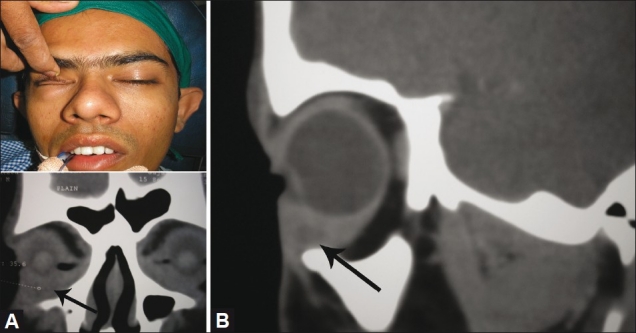
(A) (Top) fullness in the inferior orbit (bottom) showing a well-defined, hypodense, peripheral rim-enhancing lesion in relation to the mid and anterior aspect of the right inferior rectus muscle with perifocal fat stranding. (B) The sagittal reconstruction shows a ring-enhancing lesion in the inferior rectus, suggestive of an abscess

A 22-year-old man presented with complaints of swelling below the RE for 3 months in December 2007. He had history of pain and redness of the eye 4 months back, which subsided spontaneously. On examination, visual acuity both eyes (BE) was 20/20. There was a palpable, ill-defined mass with a nodular surface. It became prominent on up gaze [Fig. 2A]. The inferior fornix showed tethering and congestion. Ocular motility was normal in all gazes. Posterior segment was normal. CT scan showed a hypodense, rim-enhancing lesion in relation to the right inferior rectus muscle [Fig. 2B]. Provisional diagnosis of inferior rectus myositis was performed and the patient was prescribed oral steroid trial (1 mg/kg) for 2 weeks without any improvement. Transconjunctival inferior orbitotomy was performed and pus was drained. Gram stain and culture report were negative. Biopsy showed degenerated muscle fibers with infiltration by leukocytes. He was put on cefotaxime 1 g 12 hourly and amikacin 500 mg 12 hourly intravenously. He was discharged with oral amoxicillin plus clavulantae 625 mg bid. After 1 year of follow-up, the patient did not have any residual deformity.

### Case 3

**Figure 3:**
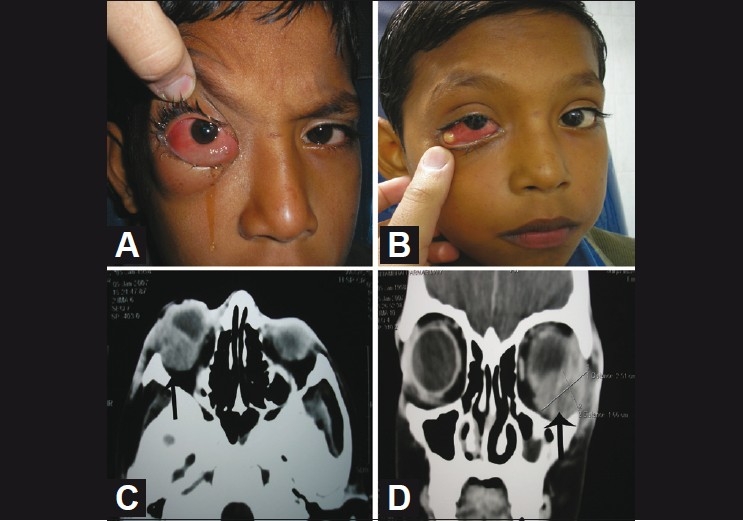
(A) chemosis and lid edema (B) showing pus pointing to the inferotemporal quadrant through the conjunctiva (C,D), showing a large ill-defined irregularly enhancing hypodense lesion in the left orbit in close relation to the lateral and inferior rectus muscle

A 10-year-old boy presented to us with complaints of sudden onset of swelling, severe pain and redness in the RE of 5 days duration in July 2006. On examination, visual acuity in both eyes was 20/20. There was edema, ptosis and chemosis. Ocular motility was restricted in down and lateral gazes [Fig. 3]. The posterior segment was normal. CT scan showed a large ill-defined, irregularly enhancing hypodense lesion in close relation to the left inferior rectus muscle. The lesion was abutting the globe in the inferolateral aspect with loss of fat plane with probably involving sclera. Provisional diagnosis of inferior rectus muscle abscess was made and the patient was started on cefotaxime 500 mg 12 hourly and gentamicin 40 mg 8 hourly intravenously. On the second day, pus was drained through the conjunctiva. Gram stain showed Gram positive cocci and the culture grew Staphylococcus. At the last follow-up (2 months), there was no residual restriction of movements.

### Case 4

**Figure 4:**
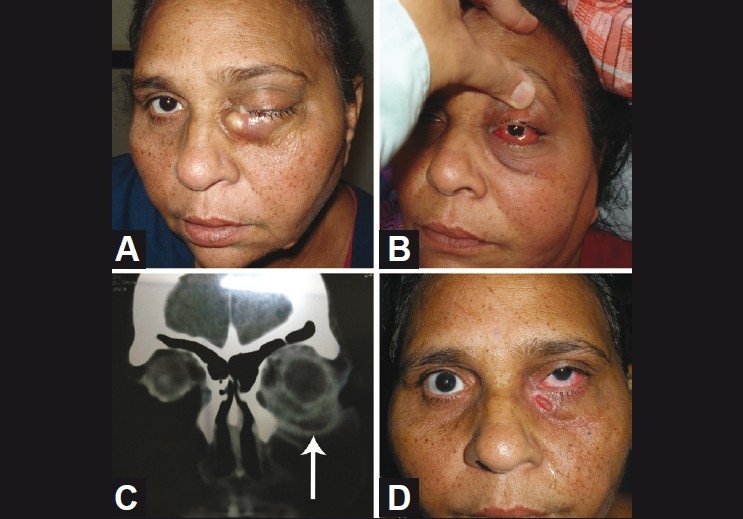
(A) proptosis, chemosis and lid edema (B) showing an irregular, ill-defined peripheral rim-enhancing hypodense lesion in the inferior rectus and inferior oblique muscle with soft tissue infiltration of the lid and cheek (C), showing pus pointing in the inferomedial quadrant through the skin (D), showing postincision and drainage clinical picture

A 55-year-old diabetic female presented to us with complaints of sudden dimness of vision, swelling, pain and redness in the LE of 5 days duration in January 2006. On examination, the pin hole visual acuity in the RE was 20/40 and there was no perception of light in the LE. There was proptosis, edema, ptosis and chemosis. The pupil was dilated and was nonreactive to light. Ocular motility was restricted in all gazes. The posterior segment showed central retinal artery occlusion. CT scan showed an irregular, ill-defined rim-enhancing hypodense lesion in relation to the inferior rectus and inferior oblique muscle with soft tissue infiltration of the lid and cheek [Fig. 4]. Provisional diagnosis of orbital cellulitis was made and ceftriaxone 1 g 12 hourly and amoxicillin plus clavulanate 1.2 g 8 hourly was started intravenously. On the second day of treatment, pus was drained through the skin. Gram stain showed Gram positive cocci and the culture grew Staphylococcus. Her vision did not improve at all. There was hypertropia of the LE and motility was restricted in down gaze at last follow-up after 14 months.

## Discussion

Of the three reported cases, two were due to *Staphylococcus aureus* and the third was idiopathic. *Staphylococcus aureus* is responsible for 95% of the cases in tropical areas and 70% of the cases of pyomyositis in nontropical areas.[4] The etiology of pyomyositis is uncertain.[4] Given the lack of adjacent orbital inflammation in our cases, we suspect that recti muscles were possibly seeded through hematogenous spread.

CT scan or magnetic resonance imaging (MRI) is required for precise localization and diagnosis. Haufschild *et al*.[2] reported an idiopathic isolated abscess of superior rectus muscle in an 11-year-old girl. MRI showed an abscess with central cavitation and a rim of enhancement. The patient was treated with intravenous antibiotics. Verma *et al*. reported two cases of young boys with EOM abscess on MRI.[3] In both the patients, ultrasonography-guided aspiration was performed and the patients treated with antibiotics. The culture grew Staphylococcus in both of them. There was no residual deformity reported in these cases. In isolated EOM pyomyositis, paranasal sinuses may be normal. Differential diagnosis should include myocysticercosis, nonspecific myositis, orbital cellulitis, dermoid cyst with infection, neuroblastoma, rhabdomyosarcoma and carotid cavernous fistula.

In conclusion, pyomyositis of EOM should be considered in any patient presenting with acute onset of orbital inflammation and characteristic CT or MRI features. Once the diagnosis is suspected, incision and drainage coupled with antibiotics eradicate the infection in most patients.
